# Small lesion–high risk: diagnostic performance of artificial intelligence in paediatric fractures with medicolegal impact

**DOI:** 10.1007/s00247-025-06456-3

**Published:** 2025-11-12

**Authors:** Johanna Pape, Oliver Johannes Deffaa, Peter Zimmermann, Martin Lacher, Franz Wolfgang Hirsch, Daniel Gräfe

**Affiliations:** 1 Department of Pediatric Radiology, University Hospital Leipzig, Liebeigstraße 20A, 04103 Leipzig, Germany; 2 Department of Pediatric Surgery, University Hospital Leipzig, Leipzig, Germany

**Keywords:** Artificial intelligence, Diagnostic accuracy, Fractures, Paediatrics, Radiography, Software

## Abstract

**Background:**

Certain paediatric fractures carry a high medicolegal risk if overlooked. While artificial intelligence (AI)-based diagnostic tools have demonstrated promising results in general fracture detection, their accuracy in identifying rare but high-risk paediatric injuries remains insufficiently studied.

**Objective:**

To assess the diagnostic performance of a commercial AI-based software (SmartUrgence, Milvue, Paris, France) in detecting a predefined set of medicolegally relevant paediatric fractures.

**Materials and methods:**

This retrospective, single-centre study analysed radiographs from 125 paediatric patients (ages 2 years to ≤17 years) with one of the following fracture types: lateral humeral condyle fractures, Monteggia fractures, trampoline fractures of the proximal tibia, or medial malleolar fractures. AI-generated results were compared against a reference standard established by two board-certified paediatric radiologists, using clinical, imaging, and intraoperative data. Sensitivity and specificity were calculated for each fracture type.

**Results:**

Sensitivity was highest for trampoline fractures of the proximal tibia (100%) and medial malleolar fractures (78%), with specificity at the knee and ankle approaching 100%. For lateral humeral condyle fractures, sensitivity reached 73%, while specificity remained high at 90%. The most significant limitation concerned Monteggia fractures: although ulnar fractures were detected with 81% sensitivity, only 2% of associated radial head dislocations were identified correctly.

**Conclusion:**

While the AI-based software demonstrated overall strong performance, its emphasis on specificity limits its utility in detecting high-risk fractures for which sensitivity is paramount. Future development should focus on enhancing sensitivity, particularly for the detection of elbow dislocations.

**Graphical abstract:**

**Figure:**
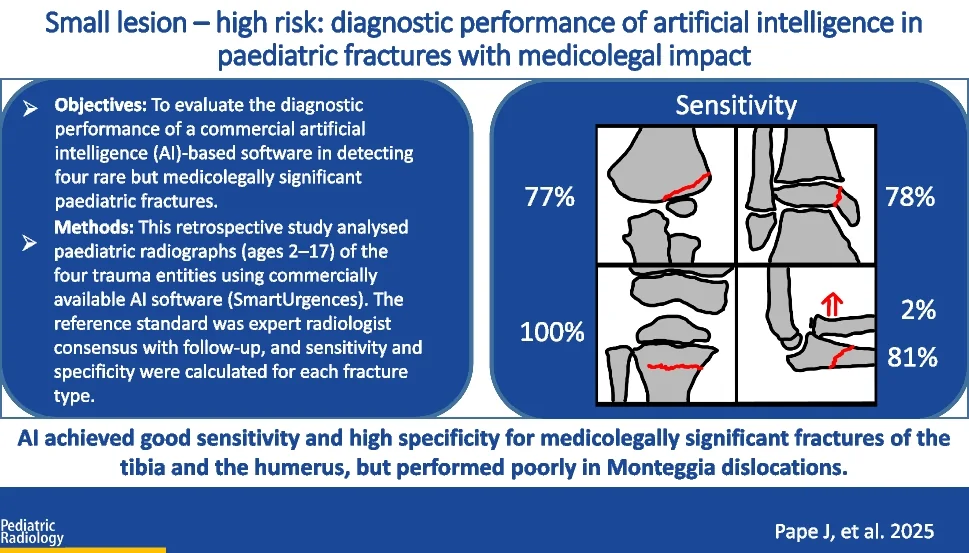

**Supplementary Information:**

The online version contains supplementary material available at https://doi.org/10.1007/s00247-025-06456-3.

## Introduction

Fractures are among the most common injuries in childhood and pose a particular diagnostic challenge in emergency settings. A distinct group of five paediatric skeletal injuries has been identified as carrying a high medicolegal risk if not promptly and accurately diagnosed [1]. Delayed recognition or suboptimal management of these injuries may lead to serious complications and could have legal ramifications, potentially necessitating formal legal defence by the treating physician.

This high-risk group includes lateral humeral condyle fractures [2, 3], traumatic radial head dislocations with associated ulnar fractures (Monteggia fractures) [4], greenstick fractures of the proximal tibial medial metaphysis (trampoline fractures of the proximal tibia) [5], medial malleolar fractures (Salter-Harris types III and IV) [6], and malaligned supracondylar humeral fractures with persistent antecurvation or rotational deformity. Each injury is characterised by distinct anatomical, radiographic, and clinical features, and accurate identification is crucial to prevent long-term morbidity.

In non-displaced lateral humeral condyle fractures, distinguishing between incomplete (and therefore stable) and complete (unstable) fractures is essential. In Monteggia fractures, correct recognition of both the ulnar fracture and the radial head dislocation is of paramount importance, as persistent radial head dislocation may lead to asymmetric growth and restricted elbow motion. Detection is particularly challenging in cases of plastic (bowing) deformities—fractures seen almost exclusively in children—where the bone bends without overt cortical disruption.

For trampoline fractures of the proximal tibia, casting in varus alignment is recommended to minimise the risk of progressive varus deformity (Cozen’s phenomenon). Medial malleolar fractures are associated with a high risk of premature physeal closure and must therefore be closely monitored to detect potential ankle deformity. These fractures often present subtly and require a high index of suspicion and careful radiographic evaluation for diagnosis [7].

The advent of artificial intelligence (AI)-based diagnostic tools has introduced new opportunities to enhance sensitivity and specificity in fracture detection. Several studies have demonstrated that state-of-the-art AI-based systems can achieve diagnostic accuracy comparable to that of experienced radiologists [8]. While not intended to replace clinical judgement, such systems may serve effectively as second readers in the assessment of most adult and paediatric fractures. However, the diagnostic performance of these tools in detecting rare, but clinically and legally significant, paediatric fractures and dislocations remains largely unexplored. Given the potential consequences of missed diagnoses in such cases, a high sensitivity is essential.

We hypothesised that the AI-based diagnostic tool would demonstrate high sensitivity, albeit with potentially variable specificity, across a defined set of medicolegally relevant paediatric fractures.

## Materials and methods

### Cohort

In this retrospective study, radiographs of patients ages 2 years to ≤17 years were included if they were acquired between 2008 and 2024 at a tertiary care centre with a dedicated paediatric surgery department, featuring a specialised paediatric orthopaedic trauma unit and an affiliated paediatric radiology institute. Eligible examinations were performed to assess suspected trauma-related injuries. Patients were identified via the radiology information system based on four specific trauma entities, as classified by van Laer: lateral condyle fractures of the humerus, Monteggia fractures, trampoline fractures of the proximal tibia, and medial malleolar fractures. Malaligned supracondylar humerus fractures were excluded, as the assessment of rotational malalignment or grading of displacement lies outside the intended scope of the AI-software evaluated.

Radiographs from external institutions were excluded, as were fractures with displacements exceeding 3 mm, as such injuries are unlikely to be missed and thus do not meet the criteria for medicolegally relevant fractures. Non-diagnostic studies—such as those with extensive cast coverage—were also excluded. All radiographs were acquired using the same digital radiography system (Axiom Aristos Fx, Siemens Healthineers, Erlangen, Germany). All radiographs were obtained under routine clinical conditions at our institution without additional preprocessing and were analysed by the AI system in their original form, acknowledging that suboptimal positioning reflects a real-world challenge.

For the control group, age- and anatomical region–matched radiographs were selected from patients imaged at the same institution between March 2023 and May 2025. These studies showed no radiographic evidence of trauma [9]. Thus, the retrospective inclusion period for the medically relevant fractures was longer than that for the control group due to the rarity of these conditions, as normal findings are much more common and because this cohort had already been processed for another study [9]. Approval for this retrospective study was obtained from the local ethics committee (357/23-EK).

### Reference standard

The reference standard was defined based on the following criteria:

- Independent evaluations by two board-certified paediatric radiologists (D.G. and F.W.H.), each with over 10 years of experience in paediatric trauma imaging
- Review of available follow-up imaging, particularly in confirmed fracture cases
- Clinical follow-up data, especially in cases initially classified as normal
- Intraoperative findings, where available (applicable to all Monteggia fracture cases)

Radiographic assessments were performed independently by the two radiologists. In cases of disagreement, final classification was determined through consensus, which was achieved for all patients.

### Artificial intelligence

All radiographs were analysed using the Conformité Européenne(*CE*)-marked AI-based software *SmartUrgence* (Milvue, Paris, France; version 2.4.0) with regard to both fractures and, at the elbow joint, dislocations. The software is designed to automatically detect and visualise skeletal trauma on conventional radiographs using deep learning algorithms trained on a multicentre dataset comprising over 120,000 annotated examinations and tested on more than 80,000 radiographs reviewed in consensus. No clinical data were provided to the software. Only imaging data were analysed.

Detected findings are categorised using internal confidence scores as follows: “YES” (high confidence), “DOUBT” (low confidence), or “NO” (no finding). Fractures are marked using bounding boxes—solid for high-confidence detections and dashed for uncertain cases. Elbow dislocations are reported as binary outcomes. The system is intended to support, but not replace, clinical decision-making.

### Statistical analysis

All fracture detections by the AI-based software were classified as positive, regardless of the confidence level assigned. In the context of Monteggia fractures, only the presence of radial head dislocation was considered the decisive pathological feature, as long-term sequelae in this injury pattern are primarily determined by the dislocation rather than the associated ulnar fracture.

Normality was assessed using the Shapiro-Wilk test. In case of non-normal distribution, data were reported as median and interquartile range. Sensitivity and specificity were calculated separately for each fracture type using standard definitions, with 95% confidence intervals (CIs) computed using the Wilson method. Accuracy, positive predictive value, and negative predictive value were not reported, as the study dataset does not reflect the true prevalence of these injuries. All statistical analyses were conducted using RStudio (version 2023.06.2, PBC), and a significance threshold of *P*<0.05 was applied.

## Results

A total of 26 patients with lateral condyle fractures of the humerus, 23 with medial malleolar fractures, 24 with trampoline fractures of the proximal tibia, and 52 with Monteggia fractures were included in the study (Table 1). There was no statistically significant age difference between the cohorts with medicolegally relevant fractures and the healthy control group (*P*>0.05).

**Table 1:** Patient demographics for each fracture type and the control group used for specificity analysis. Age is presented in years; interquartile range in parentheses

	Fractures		Controls		*P*-value
	*n*	Age	*n*	Age	
**Medial malleolus fractures**	23	13.0 (12.0–14.3)	93	12.4 (8.7–14.7)	0.26
**Trampoline fractures of the proximal tibia**	24	3.9 (3.5–4.8)	69	6.3 (3.2–11.0)	0.12
**Lateral condyle fractures**	26	5.8 (3.5–7.5)	61	5.1 (3.4–11.6)	0.48
**Monteggia fractures**	52	5.6 (4.2–7.2)	61	5.1 (3.4–11.6)	0.72

The highest sensitivity was observed for trampoline fractures of the proximal tibia, with all cases correctly identified by the AI system (100%) (Table 2). In the case of lateral humeral condyle fractures, 19 out of 26 cases (73%) were correctly classified as fractures. Notably, two of these 26 cases demonstrated secondary displacement during follow-up; both had been correctly detected by the AI system at the time of initial presentation.

**Table 2:** Diagnostic performance of the artificial intelligence-based software across selected medicolegally relevant paediatric fractures. Values shown as percentages; 95% confidence intervals in parentheses

	**Sensitivity**	**Specificity**
Medial malleolus fractures	78 (58–90)	98 (92–99)
Trampoline fractures of the proximal tibia	100 (86–100)	99 (92–100)
Lateral condyle fractures	77 (58–89)	90 (80–95)
Monteggia fractures - Ulna fracture - Radial head luxation	81 (68–89) 2 (0–10)	

In stark contrast, the AI-based software identified only 1 out of 52 radial head dislocations associated with Monteggia fractures, yielding a sensitivity of just 2%. The accompanying ulnar fractures were detected with a sensitivity of 81%. Among these, three were plastic (bowing) fractures—none of which was recognised by the AI system.

Specificity was excellent at the ankle and knee, approaching 100%, and remained high at the elbow, with a specificity of 90%. The absolute numbers of true positives, false positives, true negatives, and false negatives are presented in supplementary material 1.

**Fig. 1:**
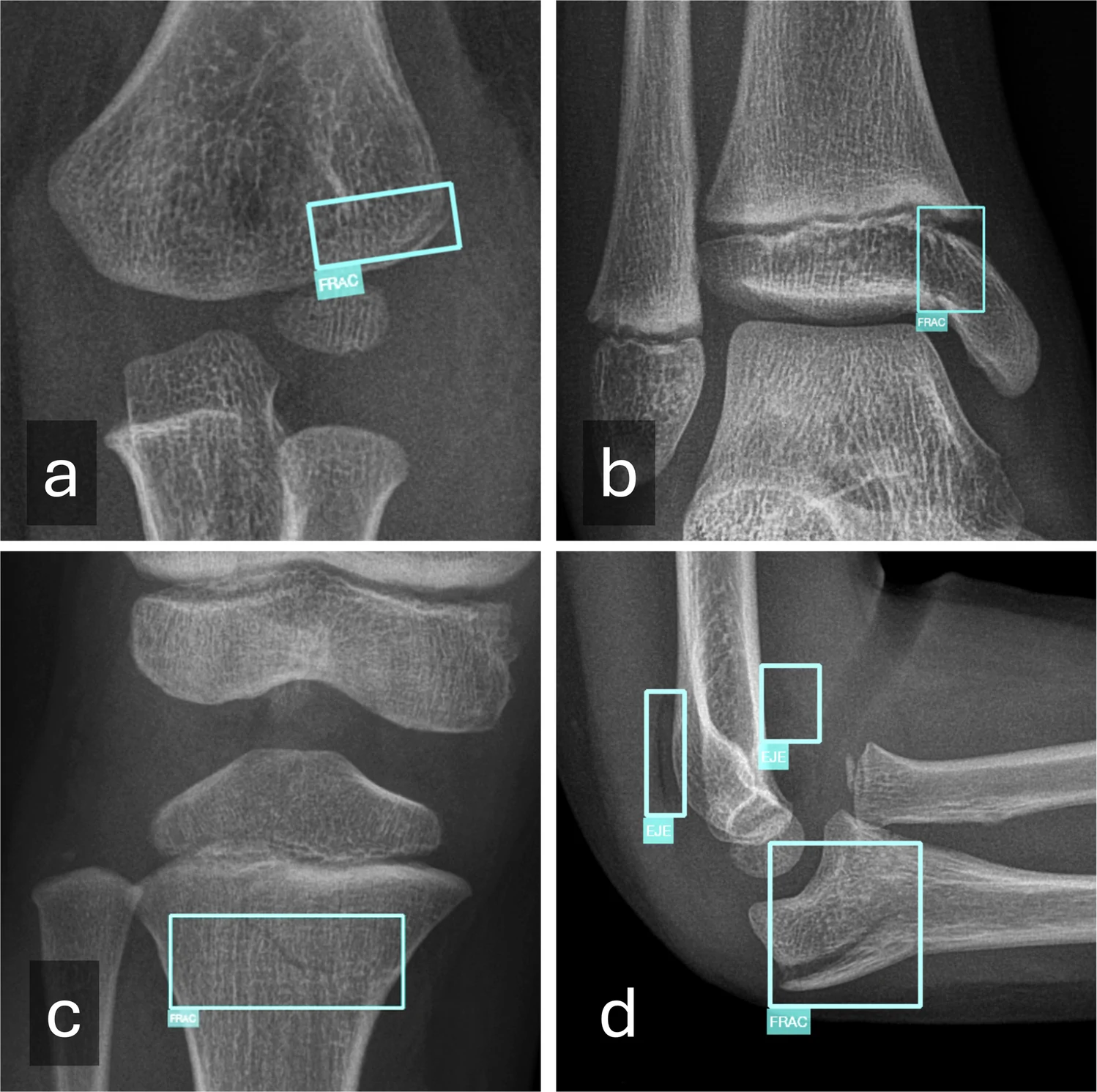
Representative radiographs of four medicolegally relevant paediatric fractures (FRAC): (**a**) lateral condyle fracture of the humerus (3-year-old girl, posteroanterior view), (**b**) medial malleolus fracture (9-year-old boy, posteroanterior view), (**c**) trampoline fractures of the proximal tibia (4-year-old boy, posteroanterior view), and (**d**) Monteggia fracture with elbow joint effusion (EJE) (5-year-old girl, lateral view). Fractures in (**a**–**c**) are correctly detected and highlighted by the artificial intelligence using bounding boxes. In (**d**), the system identifies the ulnar fracture and joint effusion but fails to detect the radial head dislocation

Representative examples of the four fracture types assessed in this study are illustrated in Fig. 1. Examples of fractures missed by the AI-based software are added in supplementary material 2.

## Discussion

This study evaluated the diagnostic performance of a *CE*-certified, AI-based software system in detecting a predefined set of medicolegally relevant paediatric fractures.

The most notable result was the system’s excellent specificity at the knee and ankle, approaching 100%. In contrast, a previous study using the same dataset reported lower specificity with an alternative AI system, RBFracture (Radiobotics, Copenhagen, Denmark): 81% (95% CI, 67–87%) for medial malleolar fractures and 89% (95% CI, 81–96%) for trampoline fractures of the proximal tibia. Even at the elbow—a complex joint during growth due to multiple ossification centres and a frequent source of false positives—specificity remained high at 90% (RBFracture, 90%; 95% CI, 83–96%).

From a clinical perspective, such high specificity is valuable, particularly in common, radiographically apparent injuries such as those of the proximal tibia and ankle. In these cases, the AI system could serve as a reliable second reader, particularly in busy emergency departments or in settings with limited radiological expertise. Accurate exclusion of fractures may help avoid unnecessary immobilisation or hospital admission, improving both workflow efficiency and patient comfort.

However, sensitivity varied considerably between fracture types. While detection was excellent for trampoline fractures of the proximal tibia (100%), it was lower for medial malleolar fractures (78%) compared to RBFracture’s 96% and 100%, respectively. The lowest sensitivity was observed in lateral humeral condyle fractures. This pattern appears consistent across different *CE*-certified AI tools, with reported sensitivities of 68% (RBFracture) and 74% (BoneView, Gleamer, Paris, France), indicating a broader diagnostic challenge for this fracture type.

This trade-off, high specificity at the expense of sensitivity, is problematic for medicolegally relevant injuries, where failure to detect subtle fractures may lead to long-term complications. A sensitivity of only 73% is insufficient for making safe discharge decisions in the absence of clinical oversight. Nevertheless, in our cohort, the two cases of lateral condyle fractures that progressed to secondary displacement had both been correctly identified by the AI system at the initial presentation.

The most critical limitation of the AI system was its near-total failure to detect Monteggia fractures—characterised by a proximal ulnar fracture combined with radial head dislocation. While ulnar fractures (excluding bowing types) were detected with a sensitivity of 81%, the dislocated radial head—a key determinant of long-term outcome—was identified in only 2% of cases. Both subtle and overt dislocations were missed, suggesting these injuries were likely underrepresented in the system’s training data.

This is particularly concerning given the clinical significance of Monteggia fractures, which are considered “don’t-miss” injuries in paediatric trauma. Their detection remains challenging even for experienced clinicians. AI systems must be specifically trained to recognise these injuries, despite their low prevalence.

To improve detection of such rare but high-stakes injuries, future research should focus on targeted AI-training using curated datasets that include underrepresented paediatric fracture patterns. Additionally, prospective studies are needed to evaluate how AI affects human diagnostic performance and, ultimately, patient outcomes. According to van Leeuwen’s evidence framework, this study represents a level II (standalone performance) evaluation [15]. Higher-level evidence—such as reader studies or outcome-based assessments—is essential to fully establish the clinical utility of these tools.

Advanced systems might also integrate patient age, trauma mechanism, or clinical data to provide context-aware decision support. In everyday practice, radiographic interpretation is rarely made in isolation. A system capable of flagging high-risk fracture constellations could increase diagnostic vigilance, particularly in subtle or easily overlooked injuries.

Several limitations of this study must be acknowledged. The retrospective, single-centre design and the relatively small sample size (23–52 cases per fracture type) and exclusion of external radiographs limit generalisability and contribute to wide confidence intervals. The synthetic dataset introduces selection bias, as it does not reflect the true prevalence of injuries. In addition, the 3 mm displacement threshold for exclusion of obvious fractures is somewhat arbitrary, given the lack of established evidence on diagnostic thresholds. Furthermore, the next higher level of evidence according to van Leeuwen, namely the assessment of the influence of AI outputs on the diagnostic performance of human readers, was not addressed. Consequently, it remains uncertain whether human readers commit errors comparable to or different from those of the AI. Moreover, the true impact is particularly difficult to evaluate for rare fracture types. Even in very large control cohorts, the artificially increased prevalence of such fractures would inevitably focus readers’ attention on the corresponding regions, thereby producing an artificially elevated and unrealistic sensitivity.

Importantly, radiographs of Monteggia fractures typically include both elbow and forearm views. The AI system categorised these images as “forearm” rather than “elbow,” likely contributing to the failure to detect radial head dislocations. From a clinical perspective, such technical classifications are secondary to the practical implication: the dislocations were missed.

## Conclusion

In conclusion, the AI-based software demonstrated good performance as a second reader for most medicolegally relevant paediatric fractures, with some limitations in detecting lateral humeral condyle fractures. However, its performance in identifying Monteggia fractures—where accurate detection is most critical—was inadequate. Clinicians should therefore continue to assess radial head alignment in all cases of isolated ulnar fractures. While AI-based software cannot yet replace clinical judgement in high-stakes scenarios, with targeted development and validation, it may become a reliable tool for enhancing paediatric fracture detection.

## Supplementary Information

Below is the link to the electronic supplementary material.

ESM 1 Supplementary File 1 (DOCX 15.3 KB)

ESM 2 Supplementary File 2 (DOCX 16.2 KB)

ESM 3 (PNG 1.38 MB)

High resolution image (TIF 4.33 MB)

ESM 4 (PNG 918 KB)

High resolution image (TIF 3.18 MB)

## Data Availability

The datasets generated and analysed during the current study are available from the corresponding author on reasonable request.

## References

[1] von Laer L, Veigel B, Illian C (2012) Die Kadiläsionen. Trauma Berufskrankh 14:247–254

[2] Tan SHS, Dartnell J, Lim AKS, Hui JH (2018) Paediatric lateral condyle fractures: a systematic review. Arch Orthop Trauma Surg 138:809–817 29574555 10.1007/s00402-018-2920-2

[3] Wirmer J, Kruppa C, Fitze G (2012) Operative treatment of lateral humeral condyle fractures in children. Eur J Pediatr Surg 22:289–294 22570126 10.1055/s-0032-1308709

[4] Cao S, Dong ZG, Liu LH et al (2022) Missed Monteggia fractures in children treated by open reduction of the radial head and corrective osteotomy of the ulna. Sci Rep 7:16819 10.1038/s41598-022-21019-4 PMC9546922 36207388

[5] Gowtam DS, Garud AB, Sharma R, Nikam M (2021) Cozens phenomenon of proximal tibia. Int J Orthop Sci 1:339–342

[6] Luhmann SJ, Oda JE, O’Donnell J, Keeler KA et al (2012) An analysis of suboptimal outcomes of medial malleolus fractures in skeletally immature children. Am J Orthop 41:113–116 22530207

[7] Birt M, Vopat B, Schroeppel P et al (2018) Diagnosis and management of McFarland fractures. Am J Emerg Med 36:527.e5–527.e7 29237543 10.1016/j.ajem.2017.12.023

[8] Ashworth E, Allan E, Pauling C et al (2025) Artificial intelligence (AI) in radiological paediatric fracture assessment: an updated systematic review. Eur Radiol 35:5264–5286 40063108 10.1007/s00330-025-11449-9

[9] Ziegner M, Pape J, Lacher M et al (2025) Real-life benefit of artificial intelligence-based fracture detection in a pediatric emergency department. Eur Radiol 35:5881–5890 40192806 10.1007/s00330-025-11554-9 PMC12417293

[10] Guermazi A, Tannoury C, Kompel AJ et al (2022) Improving radiographic fracture recognition performance and efficiency using artificial intelligence. Radiology 302:627–636 34931859 10.1148/radiol.210937

[11] Altmann-Schneider I, Kellenberger CJ, Pistorius SM et al (2023) Artificial intelligence-based detection of paediatric appendicular skeletal fractures: performance and limitations for common fracture types and locations. Pediatr Radiol 15:136–145 10.1007/s00247-023-05822-3 PMC10776701 38099929

[12] Mathur N, Lau KK (2020) Monteggia fracture: an easy fracture to miss. Emerg Radiol 27:377–381 32086608 10.1007/s10140-020-01763-8

[13] Geis JR, Brady AP, Wu CC et al (2019) Ethics of artificial intelligence in radiology: summary of the joint European and North American multisociety statement. Radiology 293:436–440 31573399 10.1148/radiol.2019191586

[14] Brady AP, Allen B, Chong J et al (2024) Developing, purchasing, implementing and monitoring AI tools in radiology: practical considerations. A multi-society statement from the ACR, CAR, ESR, RANZCR & RSNA. Insights Imaging. 15:16 10.1186/s13244-023-01541-3 PMC10800328 38246898

[15] van Leeuwen KG, Schalekamp S, Rutten MJCM et al (2021) Artificial intelligence in radiology: 100 commercially available products and their scientific evidence. Eur Radiol 31:3797–3804 33856519 10.1007/s00330-021-07892-z PMC8128724

