# Supplementary material for: Small lesion–high risk: diagnostic performance of artificial intelligence in paediatric fractures with medicolegal impact
Source: Pediatr Radiol. 2025 Nov 12;56(9):2078–83. doi: 10.1007/s00247-025-06456-3 (PMC13546147; doi:10.1007/s00247-025-06456-3)
Supplement: Supplementary file 1 [file 247_2025_6456_MOESM1_ESM.docx]

**Supplementary material 1:** Total number of cases, absolute numbers of true positives, false positives, true negatives, and false negatives for each fracture type

|  | *n* | *True positive* | *False positive* | *True negative* | *True negative* |
| --- | --- | --- | --- | --- | --- |
| **Medial malleolus fractures** | 116 | 18 | 2 | 91 | 5 |
| **Trampoline fractures of the proximal tibia** | 98 | 24 | 1 | 68 | 0 |
| **Lateral condyle fractures** | 87 | 20 | 6 | 55 | 6 |
