# Supplementary material for: Small lesion–high risk: diagnostic performance of artificial intelligence in paediatric fractures with medicolegal impact
Source: Pediatr Radiol. 2025 Nov 12;56(9):2078–83. doi: 10.1007/s00247-025-06456-3 (PMC13546147; doi:10.1007/s00247-025-06456-3)
Supplement: Supplementary file 2 [file 247_2025_6456_MOESM2_ESM.docx]

**Supplementary material 2:** Representative radiographs of medicolegally relevant fractures not detected by the artificial intelligence system: **a** missed fracture of the medial malleolus in a 12-year-old girl (posteroanterior view), **b** missed lateral condyle fracture of the humerus in a 3-year-old boy (posteroanterior view)

**Supplementary material 3:** Sensitivity of the artificial intelligence-based fracture detection software for **a** lateral condyle fracture of the humerus, **b** medial malleolar fractures, **c** trampoline fractures of the proximal tibia, and **d** Monteggia fractures (separately assessed for ulnar fracture and radial head dislocation)
